# The AST-120 Recovers Uremic Toxin-Induced Cognitive Deficit via NLRP3 Inflammasome Pathway in Astrocytes and Microglia

**DOI:** 10.3390/biomedicines9091252

**Published:** 2021-09-17

**Authors:** Lung-Chih Li, Wei-Yu Chen, Jin-Bor Chen, Wen-Chin Lee, Chiung-Chih Chang, Hong-Tai Tzeng, Chiang-Chi Huang, Ya-Jen Chang, Jenq-Lin Yang

**Affiliations:** 1 Division of Nephrology, Department of Internal Medicine, Institute for Translational Research in Biomedicine, Kaohsiung Chang Gung Memorial Hospital, Chang Gung University College of Medicine, Kaohsiung 83301, Taiwan; longee01@gmail.com (L.-C.L.); leewenchin@gmail.com (W.-C.L.); herme381981@gmail.com (C.-C.H.); 2 Institute for Translational Research in Biomedicine, Kaohsiung Chang Gung Memorial Hospital, Kaohsiung 83301, Taiwan; wychen624@cgmh.org.tw (W.-Y.C.); htay11@cgmh.org.tw (H.-T.T.); 3 Department of Neurology, Kaohsiung Chang Gung Memorial Hospital, Chang Gung University College of Medicine, Kaohsiung 83301, Taiwan; chenjb1019@gmail.com (J.-B.C.); neur099@cgmh.org.tw (C.-C.C.); 4 Institute of Biomedical Sciences, Academia Sinica, Taipei 11529, Taiwan; yajchang@ibms.sinica.edu.tw

**Keywords:** chronic kidney disease, cognitive impairment, indoxyl sulfate, inflammation, NLRP3, astrocyte

## Abstract

Chronic kidney disease (CKD) is characterized by the progressive loss of renal function; moreover, CKD progression commonly leads to multiple comorbidities, including neurological dysfunction and immune disorders. CKD-triggered neuroinflammation significantly contributes to cognitive impairment. This study aimed to investigate the contribution of uremic toxins to cognitive impairment. Serum creatinine, blood urea nitrogen (BUN), indoxyl sulfate (IS), and p-cresol sulfate (PCS) levels were measured using an enzyme-linked immunosorbent assay and high-performance liquid chromatography. The creatinine, BUN, IS, and PCS levels were increased from 4 weeks after 5/6-nephrectomy in mice, which suggested that 5/6-nephrectomy could yield a CKD animal model. Further, CKD mice showed significantly increased brain and serum indoxyl sulfate levels. Immunohistochemistry analysis revealed hippocampal inflammation and NLRP3-inflammasomes in astrocytes. Further, the Y-maze and Morris water maze tests revealed learning and memory defects in CKD mice. AST-120, which is also an IS absorbent, effectively reduced serum and hippocampal IS levels as well as reversed the cognitive impairment in CKD mice. Additionally, NLRP3-knockout mice that underwent 5/6-nephrectomy showed no change in cognitive function. These findings suggested that IS is an important uremic toxin that induces NLRP3 inflammasome-mediated not only in microglia, but it also occurred in astrocytic inflammation, which subsequently causes cognitive impairment.

## 1. Introduction

Chronic kidney disease (CKD) is characterized by the gradual and progressive loss of kidney function over months or years. CKD can be caused by diabetes, hypertension, cardiovascular heart diseases, glomerulonephritis, polycystic kidney disease, and aging. The prevalence of CKD is an important public health issue since it has resulted in a heavy burden on health care systems worldwide [1]. Patients with CKD commonly present with multiple comorbidities, including neurological disorders [2], anemia [3], cardiomyopathy and hypertension [4], secondary hyperparathyroidism [5], and immune disorders [6]. Cognitive impairment often occurs in all CKD stages, including end-stage renal disease; moreover, it affects daily life, as well as results in a higher mortality rate and longer hospitalization [7]. The prevalence of cognitive impairment in patients with CKD has been estimated to be 30–60%, which is twice as high as the prevalence in matched controls [2]. Patients with CKD present with impaired cognitive function, including memory, attention, executive function, and visual spatial function [8,9]. CKD progression leads to increased brain and serum levels of uremic toxins. However, the effects and types of uremic toxins involved in CKD-related cognitive impairment remain unclear. Among the various uremic toxins, indoxyl sulfate (IS) and P-cresol sulfate (PCS) are considered to be significantly involved in inducing cerebral–renal dysfunction [10].

Further, patients with CKD present with systemic chronic inflammation, which promotes neuroinflammation in brain tissue and causes cognitive impairment. NACHT domain-, leucine-rich repeat-, and pyrin domain-containing protein 3 (NLRP3, also known as NALP3, or cryopyrin) inflammasomes are multiple multi-protein cytosolic complexes containing pattern recognition receptors that can sense both exogenous pathogen- and damage-associated molecular patterns. Upon detection of danger signals, including uremic toxins, NLRP3 recruits and assembles a complex with an apoptosis-associated speck-like protein containing caspase recruitment domain (ASC) and caspase-1 to form the NLRP3 inflammasome, which activates caspase-1, IL-1β, and IL-18 maturation [11], and pyroptosis. IL-1β and IL-18 are crucial for physiological function in cognition, memory, and learning processes [12]. Inflammasome promotes neuroinflammation in numerous neurodegenerative disorders, including Alzheimer’s disease, Parkinson’s disease, and amyotrophic lateral sclerosis [13,14,15]. However, the role of NLRP3 inflammasome in CKD-related cognitive impairment remains unclear.

AST-120 (Kremezin, Kureha Corporation, Tokyo, Japan) is an orally administered spherical carbon adsorbent for uremic toxins and precursors, including indole and phenol groups, which are excreted through feces, resulting in the decreased serum levels of IS and PCS. AST-120 can mitigate glomerulosclerosis and increase survival in CKD animal models [16,17]. Moreover, it can attenuate uremic symptoms and delay the deterioration rate of renal function [18,19]. However, it remains unclear whether uremic toxins, especially IS and PCS, contribute to cognitive impairment. This study aimed to investigate the contribution of uremic toxins to cognitive impairment.

## 2. Materials and Methods

### 2.1. CKD Mouse Model and AST-120 Treatment

**Figure 1:**
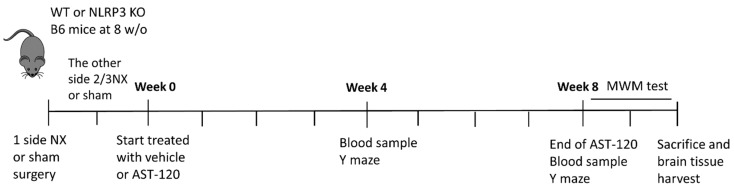
The flowchart indicating each time-point of experiment and behavioral test. Eight-week-old male C57BL/6J mice (or NLRP3 knockout mice) received sham surgery or 2-step 5/6 nephrectomy (NX) 2 weeks before AST-120 treatment. At week 0, mice were divided and treated with or without 10% AST-120. Blood was drawn from the tail vein at week 4 and week 8. Y maze tests were performed on week 4 and week 8. Morris water maze (MWM) tests were performed start from week 8. Mice were sacrificed at around week 10 and brain tissues were harvested for uremic toxin quantification, Western blotting and staining. NX: nephrectomy; WT: wild type; each interval of chart is a week.

Adult male C57BL/6J mice were purchased from the Taiwan National laboratory animal center (NLAC, Taiwan) and maintained at a 12-h light/dark cycle at 25 °C and 40–70% relative humidity with free access to food and water. Eight-week-old mice were randomly divided into the sham-control and CKD groups. CKD was induced through two-step 5/6 nephrectomy (5/6 NX); specifically, unilateral nephrectomy and 2/3 electrocoagulation of the contralateral renal cortex under anesthetization. Briefly, the 6 O surgery suture was tied around each pole of 1/3 of the left kidney and ligated. The 1/3 section of the kidney at each end was subsequently excised. After one week, the right kidney was removed after the blood vessels and ureter were sutured and transected. Further, control mice underwent sham surgery. At 2 postoperative weeks (defined as week 0 as showing on Figure 1), the C57BL/6J mice were further divided into the sham-control group, sham-control with 10% AST-120 treatment group, CKD group, and CKD with 10% AST-120 treatment group. AST-120 was mixed with food and fed to the mice for up to 8 weeks. *Nlrp3^−/−^* mice were provided by Dr. Jenny P.-Y. Ting from the University of North Carolina (Chapel Hill, NC, USA). All animal care, experimental procedures, and treatments were approved by the Laboratory Animal Care and Use Committee of Kaohsiung Chang Gung Memorial Hospital (approval number 2015022301, 18 May 2015). The time-point of each experiment is demonstrated as a flowchart in Figure 1.

### 2.2. Enzyme-Linked Immunosorbent Assay

Blood samples were drawn from the tail vein of mice at week 4 and week 8 before performing behavioral tests (Figure 1). Briefly, mice were lightly anesthetized, and blood was drawn from the tail vein; subsequently, blood samples were centrifuged to separate serum and blood cells. Serum samples were immediately frozen at −80 °C until further measurement of creatinine and blood urea nitrogen (BUN) using enzyme-linked immunosorbent assay (ELISA). Serum creatinine and BUN levels were measured using Urinary Creatinine Detection Kit (B-Bridge International, Inc., Tokyo, Japan) and Urea Nitrogen Colorimetric Detection Kit (AS ONE International, Inc., Osaka, Japan), respectively. The experiments were performed according to the user’s manual of kits. The Varioskan Lux reader (Thermo Fisher Scientifics, Waltham, MA, USA) was used to read the ELISA results.

### 2.3. Y-Maze Spontaneous Alternation Test

All mice underwent the Y-maze test as described in previous study [20] at week 4 and week 8 (Figure 1). The Y-maze comprised three equal rectangular arms. Each arm was a V-shape corridor made of white PVC (38.5 cm long, 3 cm wide, and 13 cm high); moreover, the arms were oriented at 60° angles from each other. The Y-maze test was performed under moderate lighting and quiet conditions (200 lux). At the start of each trial, the mice were placed at the end of one arm and allowed to freely explore the Y-maze for 8 min. The number and sequence of arm visits were recorded using Sony 700TVL camera (Sony Corp., Kanagawa, Japan) and analyzed using Ethovision XT software (Noldus, Wageningen, The Netherlands). “Alternation” was defined as “a consecutive entry in three different arms”. The alternation score was computed as follows: “number of alternations” divided by “total number of arm visits” minus 2. A higher score was indicative of better working memory and hippocampal function.

### 2.4. Water Maze Behavioral Test

All mice underwent the Morris water maze (MWM) test as described in previous study [21] starting from week 8 (Figure 1). A white circular plastic tank (120 cm in diameter) was filled with milky water (23 ± 2 °C, white tempera paint). Next, a transparent platform (a 10-cm diameter acrylic board) was placed in the middle of the quadrant and 1.5 cm below the water surface. The platform remained in the same location for all trials. The mice were trained twice a day at 20 min intervals for 11 consecutive days. In each training trial, the mice were allowed 120 s to find the platform. Probe trials were performed on the third and seventh days after the end of the training period. The swimming path, distance, and latency time were video-tracked and recorded using a Sony 700TVL system (Sony Corp., Kanagawa, Japan) and analyzed using Ethovision XT (Noldus, Wageningen, The Netherlands). Finally, the mean escape latency was recorded as a major outcome.

### 2.5. High-Performance Liquid Chromatography Mass Spectrometry

Mouse brain tissues (10–50 mg of each sample) were homogenized using 120 μL of 100% methanol containing internal standard (PCS-d7 20 ppb; IS-d4 10 ppb), followed by incubation on ice for 10 min. Next, the samples were centrifuged at 12,000 rotations per minute at 4 °C for 30 min. The resulting supernatant was analyzed using Waters ultra-high-performance liquid chromatography coupled with Waters Xevo TQ-S MS (Waters Corp., Milford, MA, USA). Mass spectrometry (MS) was performed in negative with multiple reaction monitoring mode. Single analysis standard dissolved in a mixture of water/methanol 50:50 (*v*/*v*) was infused for tuning purposes. Subsequently, we determined the major MS/MS fragment patterns of each analyte. The optimized parameters were as follows: capillary voltage at 1.5 kV; desolvation temperature at 500 °C; source temperature at 150 °C; and gas flow at 1000 L/h. The chromatographic separation was performed on a HSS T3 C18 (100 × 2.1 mm, particle size of 1.8 μm; Waters Corp.) at 35 °C with eluent A (0.1% formic acid in H_2_O) and eluent B (0.1% formic acid in acetonitrile); moreover, the flow rate was set at 0.35 mL/min. The gradient profile was as follows: isocratic 1% eluent B, 0.5 min; linear gradient 1–40% of eluent B, 1 min; keep 40% of eluent B, 1.5 min; 40–99% of eluent B, 0.2 min; and keep 99% of eluent B, 1 min. Next, the column was re-equilibrated with eluent A for 3.3 min. Quality-control samples were prepared and analyzed during the analytical runs after every 8th eight sample.

### 2.6. Western Blotting

Harvested brain tissues were lysed using PIRA buffer (Sigma-Aldrich, St. Louis, MO, USA) with 1X protease inhibitor cocktail (Roche, Basel, Switzerland) following the manufacturer’s instructions. The following primary antibodies were used to identify target proteins: anti-IL-1β (1:1000; Santa Cruz, Dallas, TX, USA), IL-18 (1:1000; Santa Cruz), NLRP3 (1:500; Sigma-Aldrich), caspase-1 (1:500; Santa Cruz), and GAPDH (1:2000; Santa Cruz). Various secondary horseradish peroxidase-conjugated antibodies (Millipore, Billerica, MA, USA) were used in a dilution of 1:2000–5000. Chemiluminescence on target proteins triggered using the ECL advanced system (GE Healthcare, Little Chalfont, UK). Chemiluminescent images of blots were obtained and analyzed using the UVP ChemStudio PLUS Touch system (Analytik Jena, Jena, Germany).

### 2.7. Immunohistochemistry Staining

The experimental animals were anesthetized using 5% isoflurane and perfused with 50 mL of cold phosphate-buffered saline (PBS) to purge blood from tissues. Next, 25 mL of 4% paraformaldehyde (in PBS) was perfused immediately after PBS to fix the tissues. Brain tissues were obtained after perfusion and soaked in 20% sucrose at 4 °C until they sank to the bottom. The fixed brains were dehydrated by submerging with serial alcohol concentration from 50–100%, then embedded in paraffin by Leica EG1150H paraffin embedding station (Leica Microsystem Inc, Buffalo Grove, IL, USA). The paraffin-embedded brain was coronally dissected into 10-micrometer-thick brain slices by microtome (Leica RM2235, Leica Microsystem Inc., Buffalo Grove, IL, USA). The tissue slices were used for further chromogenic and fluorescent detection.

#### 2.7.1. Chromogenic Detection

The obtained brain slices were used to examine target proteins using Goat/Rabbit HRP (DAB) Detection System (BioTnA Biotech, Kaohsiung, Taiwan), with staining as per the manufacturer’s instructions. Briefly, the brain slices were thoroughly covered with hydrogen peroxide for 10 min. Next, they were washed twice with PBS after incubation and again after being incubated with penetration buffer (0.1% Triton X-100 in PBS) for 30 min at room temperature. The samples were then incubated with immunoblocking buffer (within the kit) for 10 min at room temperature and washed with PBS. The primary and secondary antibodies were diluted with 1% BSA in PBS and incubated with the samples at 37 °C for an hour each. The primary antibodies used to detect target proteins and their corresponding dilution factors were as follows: IL-1β (1:200; Abcam, Cambridge, UK), IL-18 (1:250; Santa Cruz, Dallas, TX, USA), and caspase-1 (1:200; Santa Cruz). After primary and secondary antibody hybridization, the samples were washed with PBS. Subsequently, they were incubated with DAB mixture for 3 min at room temperature. Nuclei staining was performed by incubating the samples with hematoxylin solution for 1 min at room temperature. Sample slides were analyzed using Olympus fluorescent microscope (Olympus XM10, Tokyo, Japan).

#### 2.7.2. Fluorescent Detection

Brain slices were incubated with lysis buffer (1% Triton X-100, 100 mM glycine, 1% BSA, 0.7 mM EDTA) for 10 min at room temperature and with blocking buffer (10% bovine serum, 0.01% sodium azide in PBS). Next, they were incubated with primary antibodies (NLRP3, 1:200, AdipoGen, San Diego, CA, USA; Iba-1, 1:250, Cell signaling Technology, Danvers, MA, USA; GFAP, Cell Signaling Technology; ASC, 1:200; Santa Cruz, CA, USA)) for 60 min at 37 °C. Subsequently, the brain slices were rinsed three times with wash buffer (0.05 Triton-X 100 in PBS) at room temperature before incubation with conjugated secondary antibody (various Alexa fluorophores, 1:1000, Invitrogen, Waltham, MA, USA) for 60 min at 37 °C. After incubation, the slices were washed with PBS (3 × 5 min) and mounted with Prolong Gold containing 4′,6′-diamidino-2-phenylindole (Invitrogen, Waltham, MA, USA). Images of the immunostained brain slices were obtained using an Olympus Fluoview 10i confocal microscope system (Olympus, Tokyo, Japan).

### 2.8. Statistical Analyses

All data are presented as means ± standard error of the mean. Multiple comparisons were performed using one-way analysis of variance, followed by Bonferroni’s multiple comparison tests. Statistical significance was set at *p* < 0.05.

## 3. Results

### 3.1. Impaired Working Memory in 5/6 Partial Nephrectomy-Induced CKD Mice

The 5/6 NX procedure is commonly used to establish CKD animal models for studying uremic cardiomyopathy [22]. Accumulated uremic toxins in patients with CKD, including uric acid, IS, PCS, deoxyglucosone, pentosidine, guanidine, methylguanidine, interleukin 6, TNF-α, and parathyroid hormone, could contribute to renal–cerebral interaction dysfunction. However, the mechanisms underlying the contribution of each uremic toxin to the central nervous system (CNS) and cognitive impairment remain unclear [10,23]. We established the CKD mouse model using the 5/6 NX procedure and validating it by measuring the serum levels of creatinine, BUN, IS, and PCS using an ELISA and high-performance liquid chromatography (HPLC) coupled with MS (Table 1). Compared with the control group, the CKD mice showed increased serum levels of BUN, creatinine, IS, and PCS.

**Figure 2:**
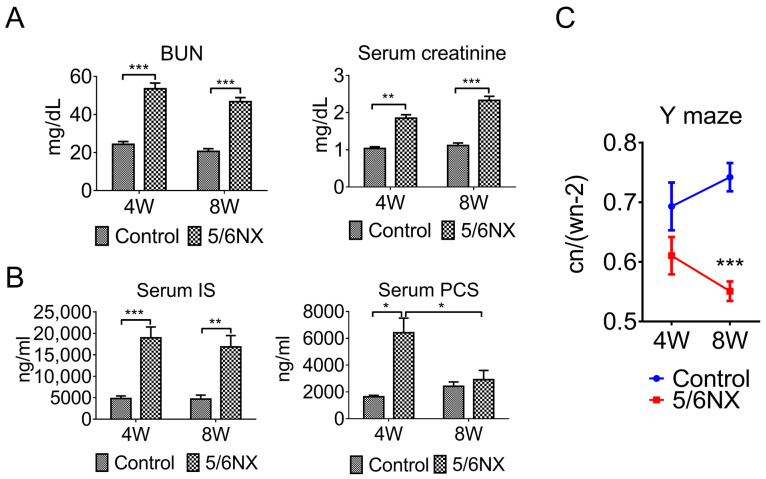
The 5/6 nephrectomy-induced chronic kidney disease (CKD) mouse models show impaired working memory. The serum levels of BUN (panel (**A**)), creatinine (panel (**A**)), and indoxyl sulfate (IS, panel (**B**)) of the 5/6 nephrectomy mice were significantly higher than those of sham-control mice at 4 and 8 weeks. However, p-cresol sulfate (PCS) levels significantly increased at week 4 but dropped back at week 8 (panel (**B**)). These findings suggest that the 5/6 nephrectomy procedure could successfully establish CKD animal models. The Y-maze behavioral test was performed at 4 and 8 weeks after the 5/6 nephrectomy procedure to examine working memory, which is also referred to as hippocampal function (panel (**C**)). The Y-maze test revealed significantly decreased cognitive function compared with the sham control group at 8 weeks after 5/6 nephrectomy surgery. Wn (walking number): number of times the animal passes different arms within 5 min; Cn (cycle number): number of times the animal goes through the three arms (n = 8, * *p* ≤ 0.1; ** *p* ≤ 0.01; *** *p* ≤ 0.001).

Specifically, there were increased serum levels of BUN, creatinine, and IS at week four and week eight; moreover, PCS levels were increased at week four but dropped close to those in the age-matched controls at week eight (Figure 2A,B). This suggests that serum PCS may not be a good biomarker for the CKD mouse model.

Naturally, mice prefer exploring new places to previously visited ones. The Y maze spontaneous alternation behavioral assay assesses the willingness of animals to investigate new arms. Spontaneous investigation tasks involve different brain regions, and this assay specifically examines cognitive function (working memory) in the hippocampus and prefrontal cortex. The Y-maze test revealed decreased cognitive function in 5/6 NX mice at week four, which significantly worsened at week eight (Figure 2C). This further demonstrated that 5/6 NX induced an accumulation of uremic toxins starting at 4 weeks post 5/6 NX; moreover, it caused cognitive impairment at week eight. Additionally, these findings suggested that the 5/6 NX procedure can be used to establish a proper mouse model for studying the CKD-induced neuropathy of the CNS.

### 3.2. Brain Levels of Indoxyl Sulfate and p-Cresol Sulfate were Increased in CKD Mice

**Figure 3:**
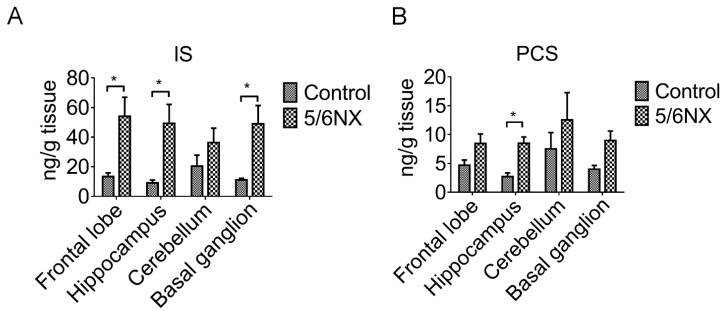
Indoxyl sulfate (IS) and p-cresol sulfate (PCS) levels were elevated in various brain regions of 5/6 nephrectomy (5/6 NX) animals compared with the levels in sham animals. Brain tissues were extracted using methanol and analyzed through high-performance liquid chromatography. There were significantly increased IS levels in the frontal lobe, hippocampus, and basal ganglia of 5/6 NX animals; however, the increase in IS levels in the cerebellum was significant (panel (**A**)). Moreover, PCS levels were increased in the various brain regions and showed a significant increase in the hippocampus compared with those in sham-control animals (panel (**B**)). These findings suggested that uremic toxins could penetrate the blood–brain barrier and accumulate in the brain of 5/6 nephrectomy animals. (* *p* ≤ 0.1; n = 4).

IS, which is also known as 3-indoxylsulfuric acid, and PCS are two small soluble metabolites of tyrosine and tryptophan, respectively, obtained from colon microbes, with >90% being bound to plasma proteins. IS and PCS are involved in various pathogeneses, including endothelial damage [24], vascular diseases [25,26], free radical production [27], and cognitive impairment [28]. However, the mechanism underlying the effect of IS and PCS on cognitive function remains unclear. We observed increased IS and PCS levels in the serum (Table 1 and Figure 1) and various brain regions of the CKD animals (Figure 3). HPLC revealed a significant accumulation of IS in the frontal lobe, hippocampus, and basal ganglion in the 8-week CKD (post 5/6 NX) animals (Figure 3A). Notably, the PCS levels were significantly increased only in the hippocampus (Figure 3B). Taken together, these findings suggest that the hippocampus is the most vulnerable brain region to uremic toxins; accordingly, we focused on this brain region.

### 3.3. AST-120 Reduced Brain and Serum Levels of Indoxyl Sulfate and p-Cresol Sulfate in CKD Mice and Reverted CKD-Induced Learning and Memory Impairment

**Figure 4:**
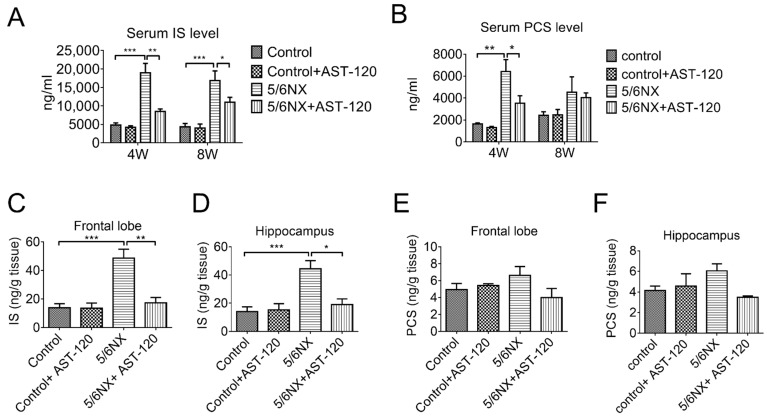
AST-120 administration decreased serum and brain levels of indoxyl sulfate (IS) and p-cresol sulfate (PCS) in 5/6 nephrectomy-induced CKD animals. Blood samples were obtained at 4 weeks and 8 weeks of experimental mice for high-performance liquid chromatography (HPLC) analysis. HPLC revealed significantly decreased serum IS and PCS levels in AST-120 treated CKD mice at 4 weeks, as well as non-significantly lower IS and PCS levels at 8 weeks compared with those in CKD mice not treated with AST-120 (panel (**A**,**B**)). Moreover, AST-120-treated CKD mice showed significantly decreased IS levels in the frontal lobe and hippocampus (panel (**C**,**D**)); however, the PCS levels in the frontal lobe and hippocampus showed a non-significant decrease compared with those in CKD mice not treated with AST-120 (panel (**E**,**F**)) (* *p* ≤ 0.1; ** *p* ≤ 0.01; *** *p* ≤ 0.00; each group contained 4–6 animals).

Our findings suggested that removing IS and PCS could effectively prevent cognitive impairment. AST-120 is an oral active carbon absorbent that binds intestinal IS and PCS and prevents their absorption into the circulation system. It is commonly used to treat patients with CKD. We administered AST-120 to the CKD animals for up to 8 weeks after 5/6 NX. The serum levels of IS were significantly decreased at 4- and 8-weeks post 5/6 NX. Moreover, the serum PCS levels were significantly decreased at 4 weeks; however, they decreased in both the CKD group and the CKD with AST-120 treated group at 8 weeks post 5/6 NX. (Figure 4A,B). Additionally, AST-120 effectively reduced IS accumulation in the frontal lobe and hippocampus (Figure 4C,D); however, it non-significantly reduced PCS accumulation (Figure 4E,F).

**Figure 5:**
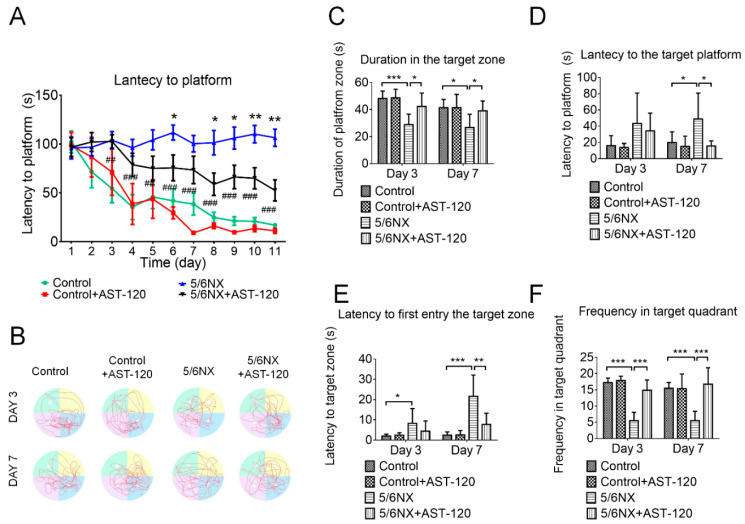
Impaired spatial learning and memory in 5/6 nephrectomy (5/6 NX) mice were prevented by AST-120 administration. Compared with control mice, 5/6 NX mice showed delayed latency in reaching the platform, which was significantly improved in AST-120-treated 5/6 NX mice (panel (**A**)). The swimming track during the probe trial at day 3 and day 7 (panel (**B**)) was recorded to determine the time duration spent in the platform quadrant, latency in reaching the platform, latency in first reaching the platform quadrant, and frequency of entering the platform quadrant. Compared with control mice, 5/6 NX mice showed a significantly shorter duration in the platform quadrant (panel (**C**)), significantly longer latency in first reaching the platform position and quadrant (panel (**D**,**E**)), and a significantly lower frequency of entering the platform quadrant (panel (**F**)) on day 3 and day 7, which was indicative of cognitive impairment. However, this impairment of learning and memory abilities was ameliorated by AST-120 administration (* 5/6 NX compared with control, ^#^ 5/6NX + 10% AST-120 compared with 5/6NX; * *p* ≤ 0.1, ** or ^##^ *p* ≤ 0.01, *** or ^###^ *p* ≤ 0.00; each group contained 5–10 mice).

To confirm altered cognitive function in CKD mice, the MWM test was used to assess whether AST-120 would prevent the impairment of spatial learning and memory. Spatial learning and memory are correlated with the synaptic plasticity of the hippocampus and frontal cortex based on reference cues for locating a submerged escape platform in an open swimming pool during training trials. In the MWM test, AST-120-treated CKD mice showed a significantly shorter latency period to reach the platform than CKD mice during the training period (Figure 5A). Additionally, the various MWM behavioral parameters were analyzed based on the swimming track (Figure 5B). The AST-120-treated CKD mice demonstrated better learning and memory abilities than the CKD animals, including a longer duration at the platform position (Figure 5C), shorter latency to reach the platform position (Figure 5D), longer duration within the target quadrant (Figure 5E), and a higher frequency of entering the target quadrant (Figure 5F). These findings suggested that AST-120 can effectively preserve learning and memory abilities in CKD animals.

### 3.4. AST-120 Suppressed Hippocampal Inflammation in CKD Mice

**Figure 6:**
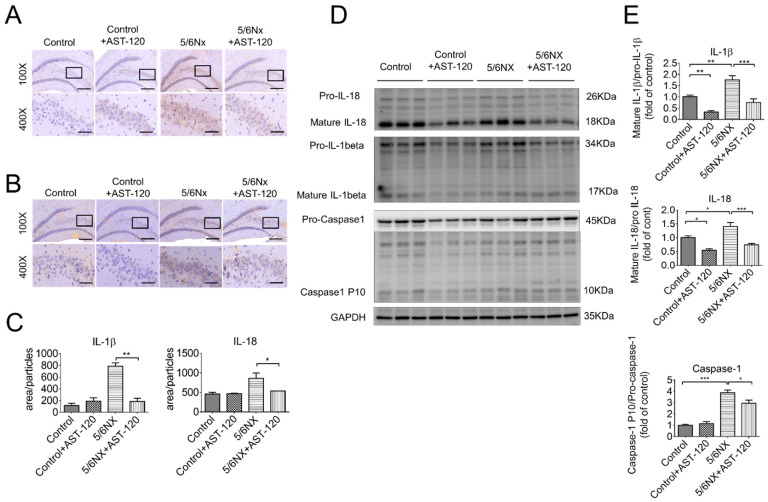
Hippocampal inflammation in CKD mice was effectively suppressed by AST-120 administration. Immunohistochemistry staining against IL-1β and IL-18 was used to examine hippocampal inflammation. There was significantly increased IL-1β (panel (**A**)) and IL-18 (panel (**B**)) expression in the hippocampal of CKD mice, which was effectively suppressed by AST-120 administration (panel (**C**)). Similarly, the Western blotting assay revealed increased IL-1β, IL-18, and caspase-1 expression in the hippocampus of CKD mice, which was reverted by AST-120 administration (panel (**D**,**E**)). The results suggested that decreased IS levels could ameliorate hippocampal inflammation. (Scale bar 100×: 200 μm, 400×: 50 μm, * *p* ≤ 0.1; ** *p* ≤ 0.01; *** *p* ≤ 0.00; n = 3 for immunohistochemistry, 6 images were taken from each animal; n = 6 for Western blotting).

The inflammatory response of the CNS, which is also termed as neuroinflammation, is the primary pathogenesis of cognitive impairment. Moreover, microglia are first-line responders to chronic or acute CNS injury, including aging and neurodegenerative diseases [29,30], traumatic brain injury [31], and ischemic stroke [32]. Therefore, we examined and compared the inflammatory state in the brains of AST-120-treated and vehicle-treated CKD mice. Immunohistochemistry images revealed that the hippocampus, especially in the Cornu Ammonis 3 (CA3) area, was positive for IL-1β (Figure 6A) and IL-18 (Figure 6B) staining in CKD mice. Moreover, the quantitative analysis revealed significantly lower IL-1β and IL-18 levels in the AST-120-treated CKD mice than in the CKD mice (Figure 6C). Further, Western blot analysis confirmed significantly lower IL-1β, IL-18, and active caspase-1 levels in the AST-120-treated CKD mice (Figure 6D,E). These findings indicated that AST-120 effectively suppressed hippocampal inflammation in CKD mice.

**Figure 7:**
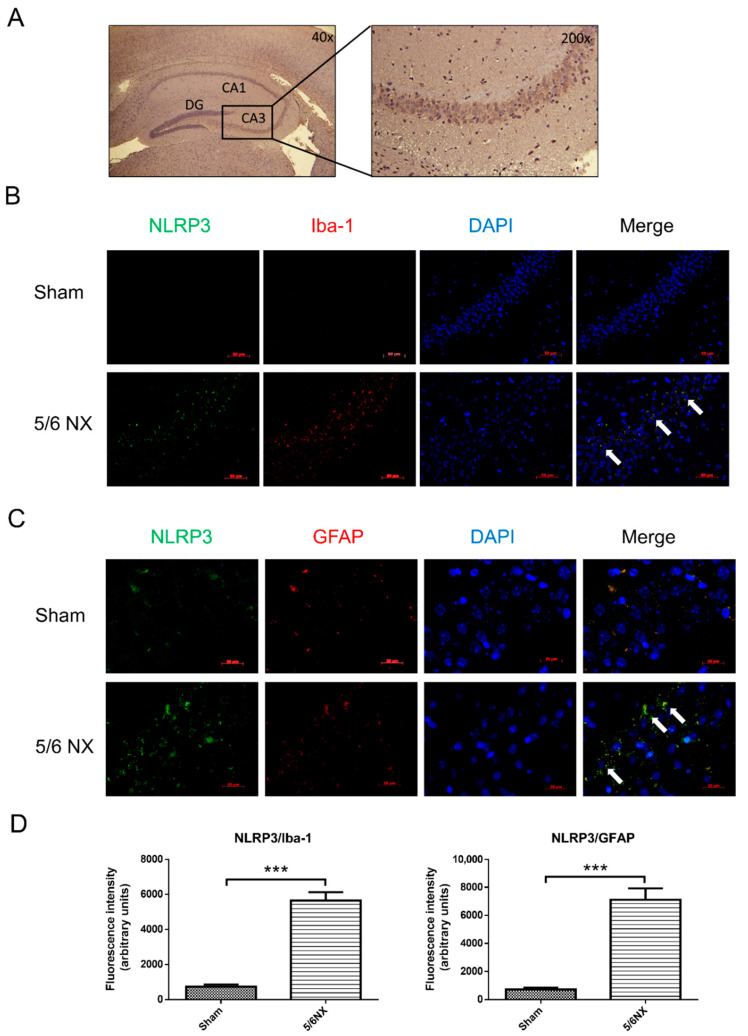
There were increased NLRP3 inflammasomes in hippocampal microglia and astrocytes of CKD mice. Bright light microscopy of hematoxylin stain demonstrated the anatomy of hippocampus with hematoxylin and eosin stain. The following images were in CA3 region of the hippocampus (panel (**A**)). The demonstration of Immunohistochemistry staining against Iba-1, GFAP, and NLRP3 was used to examine the inflammatory response in the CA3 of hippocampus in CKD mice, which demonstrated colocalization of NLRP3 foci (green, panel (**A**,**B**)) with Iba-1 (red, panel (**B**)) and GFAP (red, panel (**C**)). Additionally, the quantitative results of images demonstrated a considerable increase in NLRP3 inflammasomes in both hippocampal microglia and astrocytes of CKD mice comparing with those in sham control mice (panel (**D**)). The immunohistochemistry results suggested that hippocampal microglia and astrocytes could yield the inflammatory response to accumulated IS or PCS levels in the CKD mice. DG, dentate gyrus; CA: Cornu Ammonis. (Scale bar 50 μm; *** *p* ≤ 0.00; n = 3 for immunohistochemistry, 6 images were taken from each animal).

Our findings demonstrated that uremic toxins induce cognitive dysfunction in CKD mice. AST-120 significantly ameliorated 5/6 NX-induced IS and PCS elevation in the frontal cortex and hippocampus areas. Moreover, it reduced hippocampal inflammation, and therefore, reduced cognitive impairment. Western blot analysis revealed significantly increased hippocampal levels of the cleaved/active form of IL-1β, IL-18, and caspase-1 in CKD mice (Figure 6E). AST-120 significantly reduced inflammasome-related cytokine production in CKD and, interestingly, even in the healthy control mice (Figure 6E). The NLRP3 inflammasome was considered to be involved in the uremic toxin-triggered hippocampal inflammation. As previous results showed an increased expression of IL-1β and IL-18 in the hippocampus (Figure 6C), fluorescent immunohistochemistry was used to determine the inflammation type, which revealed that the microglia (Figure 7B, Iba-1 positive) and astrocytes (Figure 7C, GFAP positive) contained NLRP3 inflammasomes in the hippocampi of CKD mice. Immunohistochemistry revealed that uremic toxins could cause neuroinflammation, which involved both microglia and astrocytes. The quantification of immunofluorescent staining revealed that astrocytes (~7600) had higher NLRP3 positive staining than microglia (~5800) (Figure 7D), which implied that astrocytes are crucially involved in neuroinflammation.

**Figure 8:**
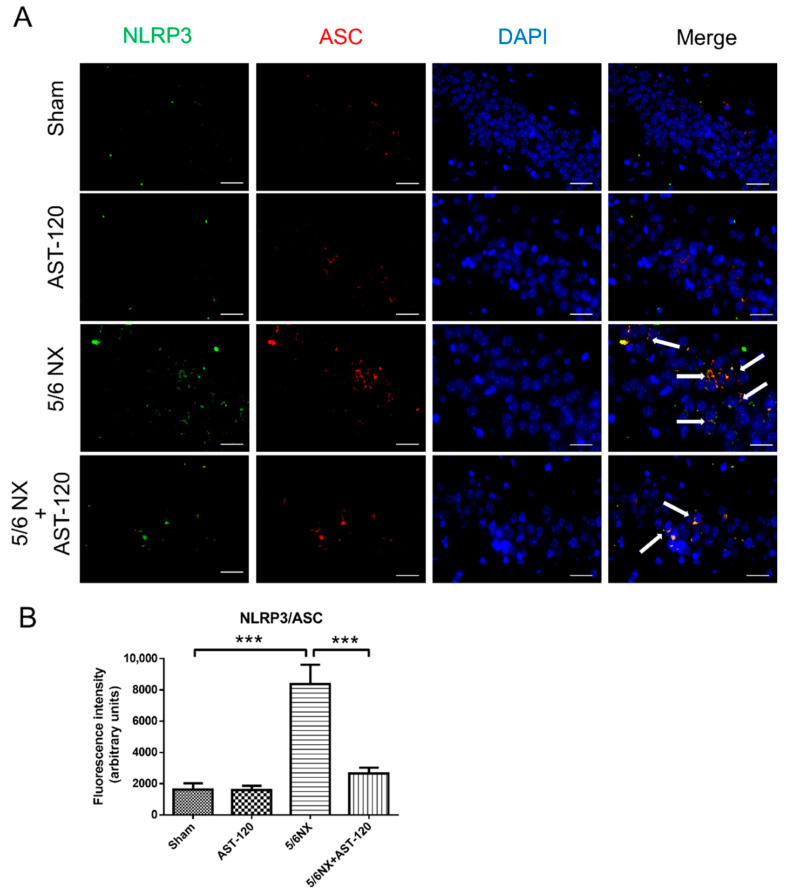
The NLRP3 inflammasome was activated in the CA3 of hippocampus of CKD mice, which was mitigated by AST-120 administration. We performed immunofluorescent staining against NLRP3 and ASC (apoptosis-associated speck-like protein containing caspase-associated recruitment domain) to examine NLRP3 inflammasome activation in the hippocampus of CKD mice, which revealed an increase and colocalization of NLRP3 (green) and ASC (red) foci in the CA3 area of hippocampus of CKD mice, which was effectively decreased by AST-120 administration (Panel (**A**)). The quantitative results of immunostaining images suggested that decreasing IS levels could reduce NLRP3 inflammasome activation in the hippocampus of CKD mice (Panel (**B**)). (Scale bar 50 μm; *** *p* ≤ 0.00; n = 3 for immunohistochemistry, 6 images were taken from each animal).

Additionally, we performed immunofluorescent staining of CA3 of hippocampal sections to examine the effect of AST-120 on neuroinflammation. The apoptosis-associated speck-like protein (ASC) is used to identify the active form of the NLRP3 inflammasome, which contains the caspase-associated recruitment domain. The quantitative results of immunofluorescent images revealed numerous active NLRP3 inflammasomes in the hippocampus of CKD mice, as well as much less active NLRP3 inflammasomes in the AST-120-treated CKD mice (Figure 8A,B). These findings suggested that IS elimination could effectively suppress NLRP3-involved neuroinflammation.

### 3.5. NLRP3-Knockout Mice Maintained Cognitive Function in CKD Mice

**Figure 9:**
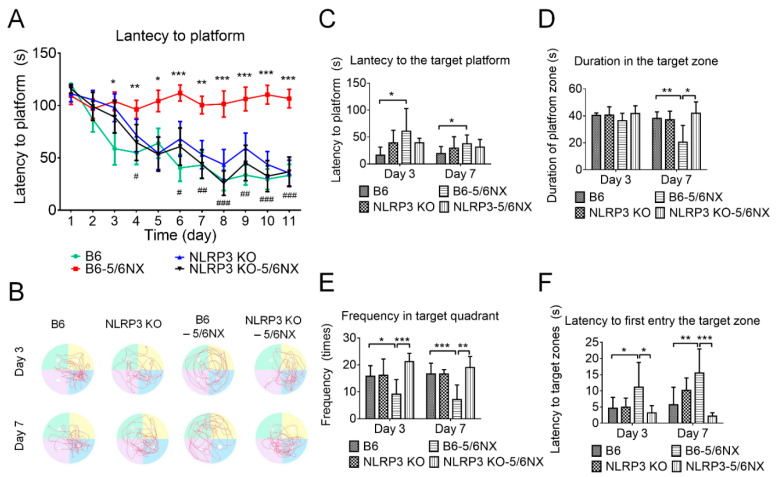
NLRP3-knockout (NLRP3 KO) mice that underwent 5/6 nephrectomy (5/6 NX) preserved better learning and memory ability than wild-type 5/6 NX-induced CKD mice. The results of the water maze training/learning period revealed that NLRP3 KO CKD mice showed similar latency in reaching the platform as control (B6) and NLRP3 KO mice, as well as a shorter latency than that in wild-type CKD mice (panel (**A**)). We recorded the swimming track of the probe trial at day 3 and day 7 after the training period (panel (**B**)) to determine the duration spent in the platform quadrant, latency in reaching the empty-platform position, latency in first reaching the platform quadrant, and frequency of entering the platform quadrant. Compared with CKD mice, NLRP3 KO CKD mice had a non-significantly shorter latency in reaching the platform position (panel (**C**)), significantly longer duration in the platform quadrant on day 7 (panel (**D**)), significantly higher frequency of entering the platform quadrant on both days 3 and 7 (panel (**E**)), and significantly shorter latency in reaching the platform quadrant on both days 3 and 7 (panel (**F**)). All the water maze results suggested that NLRP3 KO CKD mice showed resistance to CKD-induced cognitive impairment. (* 5/6 NX compared with B6 control, ^#^ NLRP3 KO+5/6NX compared with 5/6NX; * or ^#^ *p* ≤ 0.1, ** or ^##^ *p* ≤ 0.01, *** or ^###^ *p* ≤ 0.00; each group contained 8–12 mice).

Our findings suggested that NLRP3 was critically involved in IS-induced neuroinflammation. Therefore, we examined *NLRP3*-knockout (NLRP3 KO) mice. The serum levels of BUN and creatinine have shown a non-significant difference between 5/6 NX B6 and 5/6 NX NLRP3 KO mice (Figure S1), which suggested 5/6 nephrectomy induced a similar deficit of renal function in both the B6 and NLRP3 KO mice. Furthermore, the immunofluorescent image of the CA3 region of the 5/6 NX NLRP3 KO mice (Figure S2). The MWM test revealed similar cognitive function in NLRP3 KO mice as in B6 mice during the training period (Figure 9A) and probe trials (Figure 9C–F). This suggested that *NLRP3* gene knockout does not induce cognitive dysfunction in mice. Notably, compared with the CKD mice, the NLRP3 KO CKD mice had a significantly shorter latency in reaching the hidden platform during the training period (Figure 9A) and probe trials (Figure 9C), a longer duration time at the target quadrant (Figure 9D), higher frequency entering the target quadrant (Figure 9E), and shorter latency in reaching the platform position (Figure 9F). The behavioral results suggested that the NLRP3 KO mice were resistant to CKD-induced learning and memory dysfunction, as well as that NLRP3-mediated neuroinflammation was crucially involved in cognitive impairment in CKD mice.

## 4. Discussion

Our findings demonstrated that the 5/6 NX-induced CKD mice resulted in spatial working memory dysfunction. AST-120 significantly attenuated CKD-induced IS elevation in the frontal cortex and hippocampus areas, and therefore, reduced cognitive impairment. The NLRP3 inflammasome was activated in the frontal cortex and hippocampus of CKD mice; moreover, NLRP3 deficiency significantly reduced the 5/6 NX-induced spatial memory dysfunction. Our findings revealed that IS is crucially involved in NLRP3-mediated neuroinflammation and cognitive dysfunction in CKD mice.

Clinical studies have confirmed the association between uremic toxins and cognitive impairment; however, the role remains unclear. Numerous clinical studies have demonstrated that cognitive function deteriorates as the glomerular filtration rate declines [33,34]. However, long-term hemodialysis or peritoneal dialysis does not necessarily recover cognitive function [35], which implicates that uremic toxins cannot be removed through dialysis and they penetrate the blood–brain barrier (BBB) to affect brain function in patients with CKD. Both IS and PCS are protein-bound strong inducers of oxidative stress and accumulated in the brains of CKD animals [10,36]. We observed a significant increase in serum IS, but not PCS, levels with CKD progression, which suggests that serum IS may be a better marker for cognitive dysfunction in CKD mice. This is consistent with clinical reports of a negative correlation of serum IS, but not PCS, with cognitive function in patients with CKD undergoing [37] and not undergoing hemodialysis [28]. In our study, the IS levels were three times the PCS levels in various brain regions of CKD animals (Figure 3). Consistent with this finding, the brain IS levels are increased in rats with cisplatin-induced nephrotoxicity [38]. Taken together, these findings suggest that IS is more effective than PCS at interfering with BBB integrity. A recent study by Bobot et al. suggested that IS can activate aryl hydrocarbon receptors and cause BBB dysfunction in CKD rats [39]. We found that AST-120 treatment significantly reduced both the serum and brain levels of IS (Figure 4), ameliorated NLRP3 inflammasome-induced neuroinflammation in the hippocampus, and improved short-term and long-term memory impairment in CKD mice. AST-120 has been shown to reduce IS-induced glial neuroinflammation [40]. Therefore, early AST-120 treatment is a potential therapy for CKD-induced cognitive impairment. Moreover, our results not only demonstrated that AST-120 suppressed the production of active IL-1β and IL-18 in the hippocampus of CKD, but also showed reduced cleaved IL-1β and IL-18 in control mice (Figure 6D,E). The results implied that AST-120 could reduce the inflammatory response of the brain, even in healthy animals (Figure 6D,E), and slightly increased learning ability on the performance in the water maze (red line of Figure 5A). Recently, AST-120 was reported to change the microbiota in both normal and adenine-induced renal failure in mice, which may partly explain the phenomena [41].

The NLRP3 inflammasome can sense numerous danger signals and contribute to sterile neuroinflammation in multiple neurological diseases, including β-amyloid plaques and tau fibrillary tangles, which trigger the NLRP3 signaling pathway in the brains of rodent models of Alzheimer’s disease [42,43]. Similarly, the NLRP3 inflammasome was activated in the hippocampus of 5/6 NX-induced CKD mice through oligomerization with ASC (Figure 8) and subsequently produced the downstream inflammatory cytokines, IL-1 and IL-18 (Figure 6). Specifically, our study demonstrated that the expression of NLRP3 is increased in both microglial cells and astrocytes in the hippocampus of CKD mice (Figure 7). To our knowledge, this is the first study to provide in vivo evidence regarding NLRP3 inflammasome existence in astrocytes of CKD mice. Although the mechanism through which IS activates NLRP3 inflammasome remains unclear, Adesso et al. reported that IS enhanced nuclear factor-kB (NF-kB) translocation and increased ROS production in primary mouse astrocytes and mixed glial cells [44]. NF-kB may serve as an important mediator for inflammasome activation [45]. Future studies are warranted to examine the mechanisms through which IS triggers NLRP3 inflammasome activation in astrocytes and microglia and activates the downstream IL-1 and IL-18 involved in neuroinflammation.

The neuroinflammation induced by IS through NLRP3 inflammasome activation in the CA3 of the hippocampus could have contributed to the impaired working memory and spatial learning demonstrated in the MWM tests of CKD mice. A recent study by Karbowska et al. [46] showed that a higher level of IS accumulated in the brain stem and a relatively lower concentration accumulated in the hypothalamus. Although the results were slightly different from ours, probably due to different species or models; our results showed consistency that IS accumulates in different areas of the brain and leads to different neurological alterations, including memory impairment, apathetic behavior, reduced locomotor, and exploratory activity. These may be related to IS-induced inflammation or oxidative stress in different areas of the brain. It is noteworthy that inflammasome-related protein and downstream cytokines are increased in the CA3 area of hippocampus. The CA3 region has attracted major attention in recent years for its specific role in memory processes [47].

Notably, NLRP3 inflammasome deficiency prevented cognitive impairment, indicating that NLRP3 inflammasome-mediated neuroinflammation is a central driver of the progression of CKD-related cognitive dysfunction. Future studies should examine the roles of IL-1β and IL-18, two inflammasome-related cytokines, in CKD-induced cognitive impairment.

## 5. Conclusions

Our findings suggest that IS, rather than PCS, was crucially involved in CKD-induced cognitive impairment. The NLRP3 inflammasome activation in the hippocampus induced neuroinflammation and impaired short-term and long-term memory. Additionally, astrocytes were involved in neuroinflammation after IS accumulation. NLRP3 KO mice were resistant to CKD-induced cognitive dysfunction. Moreover, AST-120 improved cognitive dysfunction by eliminating IS from the circulation system and the brain. Therefore, NLRP3 inhibitors and AST-120 may be effective therapies for CKD-induced cognitive impairment.

## Figures and Tables

**Table 1:** The physiological parameters of 5/6 nephrectomy animals revealed accumulation of blood urea nitrogen (BUN), creatinine, indoxyl sulfate (IS), and p-cresol sulfate (PCS) in the circulation system. ^a^, *p* < 0.05 vs. control-4W; ^b^, *p* < 0.05 vs. control-8W.

	Sham-Operation		5/6Nx	
Post-Surgery Time	Control-4W	Control-8W	CKD-4W	CKD-8W
Animal Number	N = 8	N = 8	N = 8	N = 8
Body Weight (g)	29.5 ± 2.1	29.9 ± 0.8	25.7 ± 0.6	27.9 ± 0.8
BUN (mg/dL)	24.3 ± 5.0	20.6 ± 4.9	53.5 ± 12.3 ^a^	47.7 ± 8.6 ^b^
Creatinine (mg/dL)	1.03 ± 0.15	1.12 ± 0.23	1.85 ± 0.38 ^a^	2.35 ± 0.47 ^b^
Indoxyl sulfate (ng/mL)	5142.4 + 1660.0	4343.95 ± 2481.2	18,959.7 ± 5072.2 ^a^	18,152.5 ± 8887.5 ^b^
P-cresyl sulfate (ng.mL)	1794.9 ± 372.	2423.3 ± 900.3	6413.5 ± 2184.4 ^a^	3892.7 ± 2822.5 ^b^

## Data Availability

Not applicable.

## Supplementary Materials

The following are available online at https://www.mdpi.com/article/10.3390/biomedicines9091252/s1, Figure S1: The 5/6 nephrectomy-induced chronic kidney disease (CKD) mouse models in wild type and NLRP3 KO mice, Figure S2: The fluorescent images of immunohistochemistry have showed negative staining of NLRP3 in the CA3 region of shame and 5/6 nephrectomy NLRP KO mice.
