# Supplementary material for: The AST-120 Recovers Uremic Toxin-Induced Cognitive Deficit via NLRP3 Inflammasome Pathway in Astrocytes and Microglia
Source: Biomedicines. 2021 Sep 17;9(9):1252. doi: 10.3390/biomedicines9091252 (PMC8467651; doi:10.3390/biomedicines9091252)
Supplement: Supplementary file 1 [file biomedicines-09-01252-s001.zip › biomedicines-1326219-supplementary.pdf]

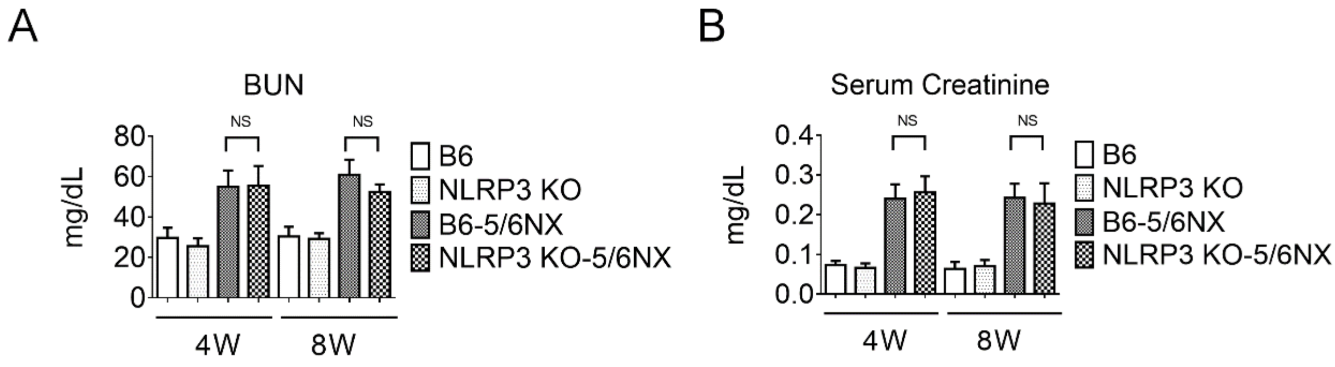


**Figure S1. The 5/6 nephrectomy-induced chronic kidney disease (CKD) mouse models in wild type and NLRP3 KO mice.** The serum levels of BUN (panel A), creatinine (panel B) of the 5/6 nephrectomy mice were significantly higher than those of sham-control mice at 4- and 8-week after 5/6 nephrectomy ($p \leq 0.001$). However, both levels of BUN and creatinine were not significantly different in 5/6 nephrectomy wild type (B6) and NLRP3 KO mice. The results suggested that 5/6 nephrectomy induced similar deficit of renal function in both B6 and NLRP3 KO mice (n=5 for each group; NS, not significant).

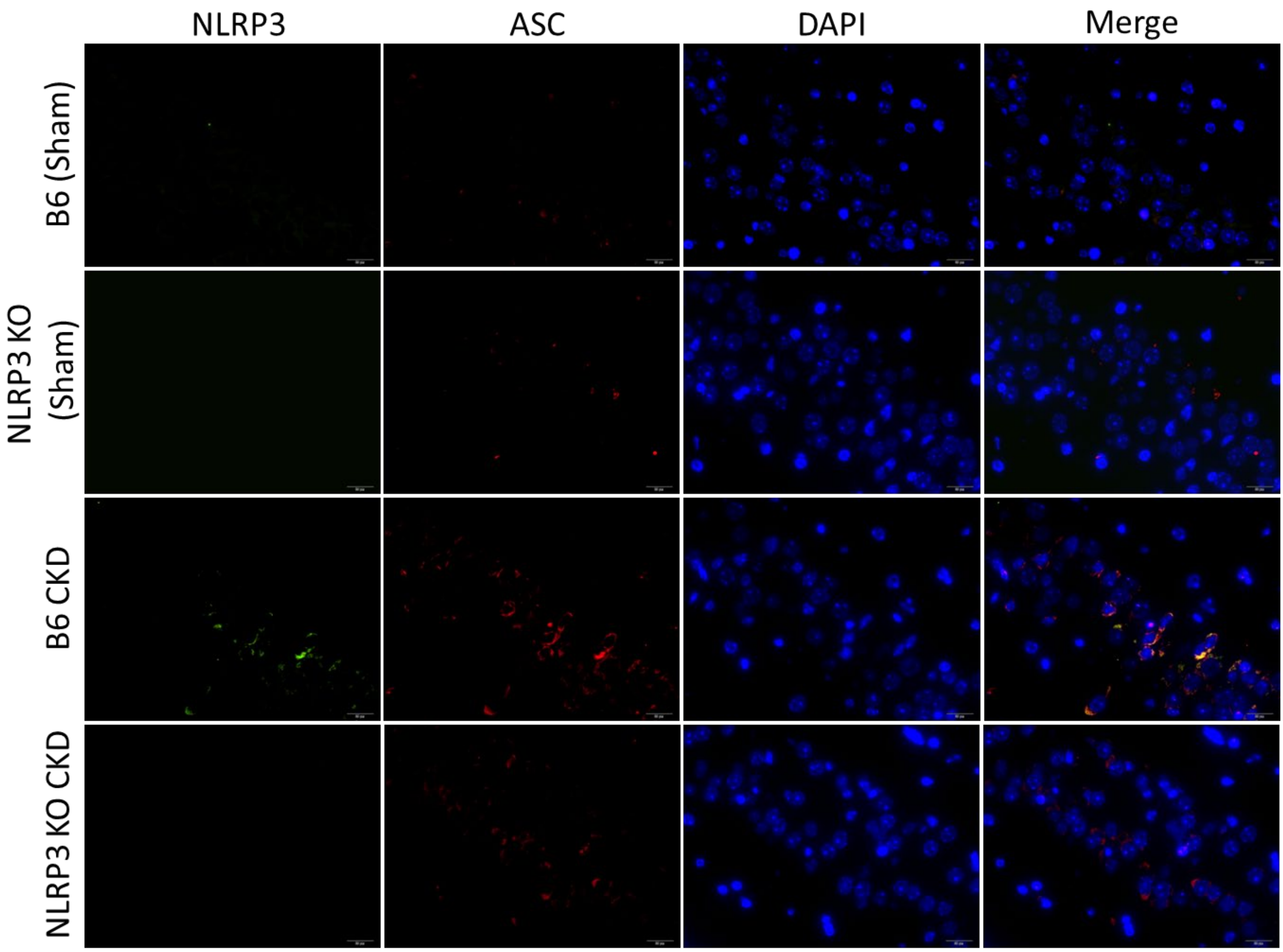


**Figure S2: The fluorescent images of immunohistochemistry have showed negative staining of NLRP3 in the CA3 region of shame and 5/6 nephrectomy NLRP KO mice.** The B6 (wild type) and NLRP3 KO sham control animals barely detected NLRP3 (red) and ASC (green) staining, which results suggested no inflammatory response occurred in CA3. The CA3 region of B6 CKD animals showed strong staining of NLRP3 and ASC, but not NLRP3 KO animals. The results indicated the *NLRP3* gene is successfully knocked out in the mice.

A

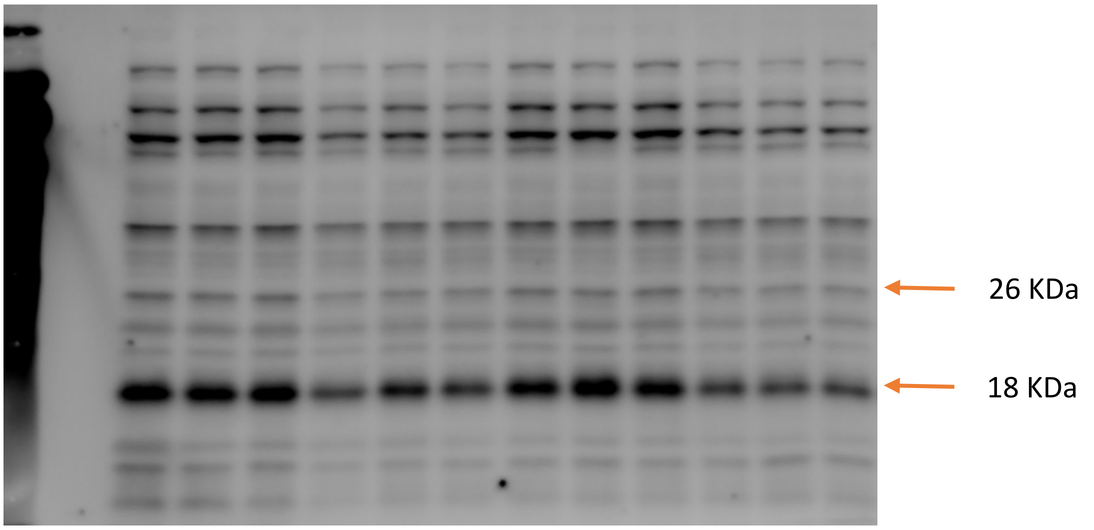


B

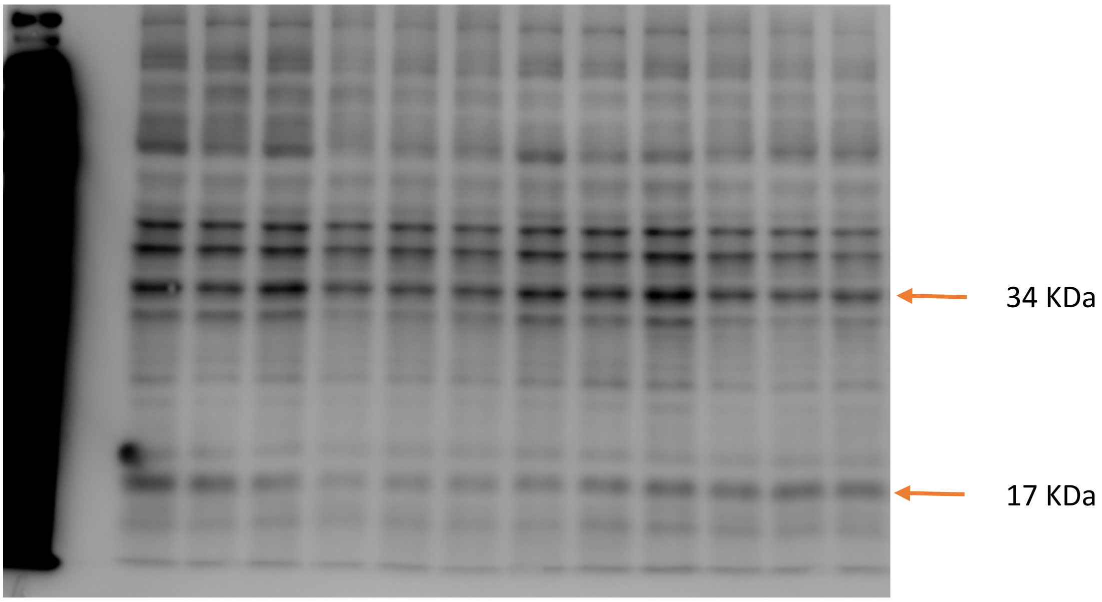

**C**

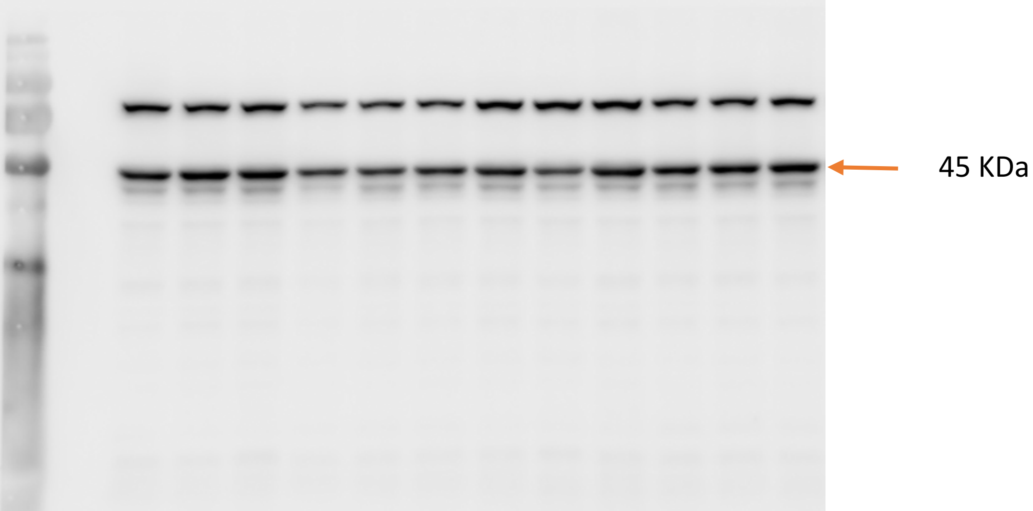


**D**

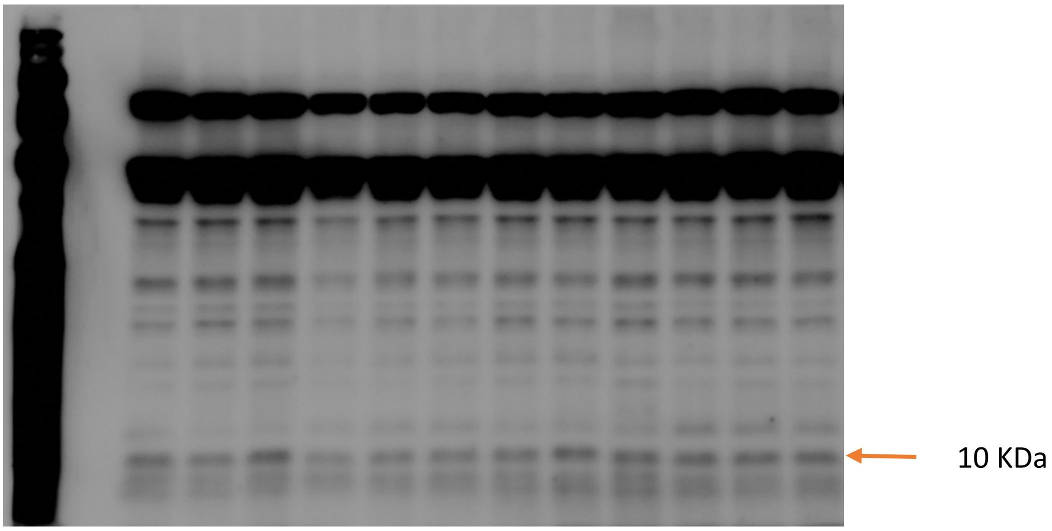


**E**

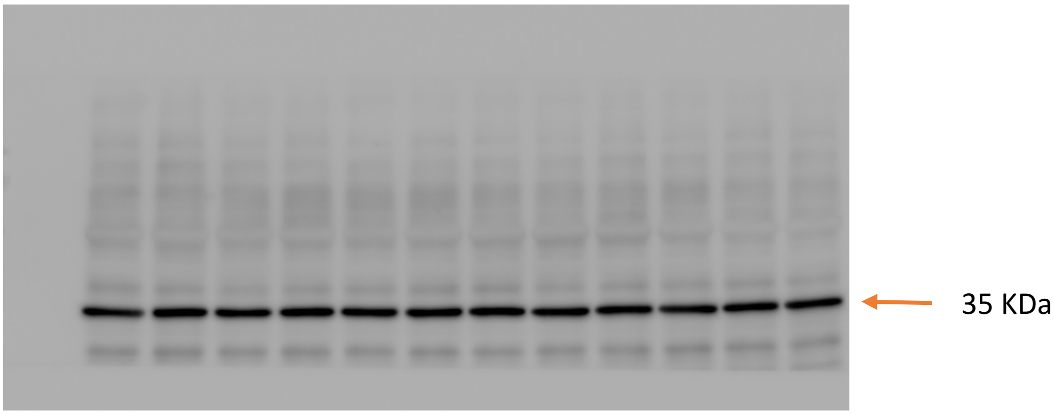


**Figure S3: The original Western blotting images of Figure 6.** (A) IL-18; (B) IL-1 beta; (C) Pro-caspase 1; (D) Caspase 1 P10; (E) GAPDH.
